# Harmful alcohol use among mothers of under-five child: findings from the Panel Study on Korean Children

**DOI:** 10.1186/s12905-021-01316-2

**Published:** 2021-04-17

**Authors:** Seulgi Kim, Seung-Ah Choe, Sung-Il Cho

**Affiliations:** 1 Department of Public Health Science, Graduate School of Public Health, Seoul National University, Seoul, 08826 Korea; 2 Department of Preventive Medicine, Korea University College of Medicine, 73 Goryeodae-ro, Seongbuk-gu, Seoul, 02841 Korea; 3 Department of Epidemiology and Health Informatics, Graduate School of Public Health, Korea University, 73 Goryeodae-ro, Seongbuk-gu, Seoul, 02841 Korea

**Keywords:** Alcohol drinking, Substance-related disorders, Mothers, Under-5 children

## Abstract

**Background:**

Harmful alcohol use in young mothers as a primary caregiver has a profound impact on their own health and interaction with the child. We studied the epidemiology of harmful alcohol use among Korean mothers and their partners of under-five children.

**Methods:**

We used a longitudinal data of 2,150 Korean mothers of the Panel Study on Korean Children (PSKC). We assessed temporal trend and risk factors for harmful alcohol use in mothers using generalized estimating equation (GEE) model. To estimate the impact of maternal harmful alcohol use on the child, the association between neurodevelopment of the child measured by the Korean-Ages & Stages Questionnaire (K-ASQ) and maternal harmful alcohol use was analyzed using GEE and generalized linear mixed model. We included mother’s age, education, preterm, year of the survey and time-varying covariates (including maternal working status, monthly household income, plan for additional childbirth, psychological stress related with childcare, current smoking, and partner’s harmful alcohol use) in the model.

**Results:**

Mean age of mothers at the baseline was 31.3 years. Annual prevalence of harmful alcohol use increased from 0.7% in the 1st year to 2.6% in the 4th year (*P* for linear trend < 0.001). Prevalence of harmful alcohol use increased by 48% per year among the mothers (adjusted odds ratio (OR) = 1.48, 95% confidence interval (CI): 1.22, 1.78). Lower education than college or university graduation was associated with higher odds of harmful alcohol use (2.52, 95% CI: 1.43, 4.45). Current smoking (7.44, 95% CI: 3.00, 18.45), harmful use of alcohol of partner (2.66, 95% CI: 1.57, 4.49) were associated with higher risk of harmful alcohol use in mothers. The association between low K-ASQ score and maternal harmful alcohol use was toward positive which did not reach statistical significance.

**Conclusions:**

In a cohort of Korean mothers participating in the PKSC, risk of harmful alcohol use increases over time and is associated with harmful alcohol use of their partners. We suggest an approach targeting both parents can be more effective in controlling the harmful alcohol use of mothers.

## Background

Harmful alcohol use is defined as alcohol consumption that results in physical or psychological harm [1]. It is a major contributor to cardiovascular disorders, gastrointestinal and/or liver problems, psychiatric diseases, and cancer [2]. Prevalence of harmful alcohol use in the general population widely ranges from < 1 to 10% [1].

Harmful alcohol use among parents of young children has life-long impacts on their own health and development of their children [3–5]. Although men are more likely to drink alcohol and drink in larger amounts, equal amounts of alcohol can cause more long-term impacts in women than men because of the biological difference in alcohol metabolism [5]. Specifically, the negative consequences of maternal alcohol consumption during pregnancy and parental alcohol consumption during childhood and adolescence on child development have been studied with a particular focus on fetal alcohol spectrum disorders (FASDs) [6]. During the postpartum including breast feeding period, harmful alcohol use was observed even in nursing women and the risk of harmful alcohol use was higher among those who became mothers in younger age [7–9]. Harmful alcohol use in mothers as a primary caregiver can also negatively affect interaction with the child and prenatal alcohol exposure of the second child [10, 11], more profoundly for the first 36 months of birth [12]. Children of alcohol-abusing parents are more likely to show alcohol or some other substance abuse and suffer from psychological disorders, poor academic performance compared to those with parents who do not abuse alcohol [13].

Given the profound impact of harmful alcohol use in the mothers, elucidating risk factors and potential targets of intervention would be important. Several maternal features including maternal precarious employment, low income, childcare-associated stress can be potential risk factors for substance use in mothers of young children which are not consistent in prior studies [6, 14–16]. In addition, pregnancy and childbirth can be a window of opportunity where women are more likely to be motivated to quit their substance abuse [8]. Using population-based longitudinal cohort data, we assessed prevalence and risk factors for harmful alcohol use among mothers of under-five children born in 2008.

## Methods

### Data

We used the Panel Study on Korean Children (PSKC) conducted by the Korea Institute of Child Care and Education which is the first national panel study on newborn babies in Korea tracking the growth process of Korean children [17]. In brief, PSKC sampled babies born in health facilities across the country from April to July 2008 and then annually collected information of childrearing environment using self-administered questionnaires for the mothers and face-to-face interviews with trained interviewers [16]. To assess the prevalence of harmful alcohol use in mothers of young children, we restricted our analysis to the data of biological mothers of under 5-year children. The first wave in 2008 included 2,150 mother-baby pairs and 81.6% of the primary sample was retained in the 4th wave study in 2011 [18]. Information about participants’ age, highest education, monthly income, employment status of the year, current smoking, harmful alcohol use and smoking of the partner, plan for additional childbirth, and parenting stress were obtained using self-administered questionnaires. Indeed, smoking has a strong association with alcohol use and vice versa [19, 20]. Because majority of the primary daytime caregivers were mothers (84%) in the PSKC [21], we focused on the epidemiology of maternal harmful alcohol use and its impact.

### Definition of harmful alcohol use and covariates

We defined high-risk alcohol drinking as an average alcohol consumption of 5 drinks or more per occasion for women and 7 drinks or more for men twice or more per week [1, 16, 22]. We selected covariates including parent’s age, education and employment status, monthly household income, harmful alcohol use and smoking, preterm birth and parenting stress scale based on previous studies [15, 16, 23]. Constant covariates were collected in 2008 survey and the answers classified into following categories: mothers’ age (< 35 and ≥ 35), highest education (≤ high school and college/university), and preterm birth as a proxy of the general health status of the baby. Annually collected information as time-varying covariates including employment status, current smoking, harmful alcohol use and current smoking of partner, plan for additional childbirth was grouped into two categories: yes or no. Unemployed included housewives and students. Monthly household income was classified in to 3 categories: < $3,442, $3,442‒6,885, ≥ $6,885. The cut-off value for household income was based on the national median household income for 4-person families which was approximately $3,442 per month in 2011 [24]. Parenting stress scale was measured pressures pertaining to parental role and distress using 10 questions which are used in a prior study [25]. The questions include, for example, the following statement: “I'm not sure if I can be a good parent”, “I'm not sure if I can raise my child well”, “Sometimes I want to run away from my child”. We created a score as a sum of the responses to each question. We calculated a sum of all responses recorded with a 5‒point Likert scale and used it as a continuous measure for childcare stress of the year.

### Assessment of the neurodevelopment of children

To estimate potential effect of the maternal harmful alcohol use on their children, we explored the association between maternal harmful alcohol use and the score of Korean-Ages & Stages Questionnaire (K-ASQ). Between 2008 and 2010, the K-ASQ were administered by the mothers using Computer-assisted personal interviewing (CAPI) [26]. The Korean version of the ASQ has been validated and standardized for Korean children, based on the ASQ [27], which assesses 5 developmental domains: communication, fine motor skills, gross motor skills, personal and social skills, and problem-solving ability. Each domain is assessed by 6 questions and scores for individual items are summed to give a continuous score for each of the 5 domains ranging between 0 and 60. The sensitivity of K-ASQ was 88% and specificity was 82.5% according to a prior report [28]. We identified infants with overall score of each domain is 2 SD below the mean which are indicative of development delay [29].

### Statistical analysis

Annual composition of covariates and prevalence of parental harmful alcohol use for the first 4 years were calculated and tested for a linear trend using a weighted logistic regression method. To compare the associated factors of harmful alcohol use between mothers and their partners, a generalized estimating equation (GEE) model was conducted accounting for the within-woman correlation of repeated measurements. This approach is to estimate the population-average effects. We calculated odds ratio (OR) and confidence intervals (CI) of harmful alcohol use among mothers and their partners adjusted for age of mother and her partner, highest education, preterm birth, year of survey, and time-varying covariates (including maternal working status, monthly household income, plan for additional childbirth, psychological stress related with childcare, current smoking and partner’s harmful alcohol use) [15, 16, 23, 25]. To estimate potential impact of maternal harmful alcohol use, adjusted ORs of low score of K-ASQ (2 SD below the mean) for maternal harmful alcohol use were calculated. In further analyses, we fitted generalized linear mixed models calculating least-squares means of K-ASQ score. The score for each K-ASQ domain was converted into standardized value (z-score). Maternal current smoking was not included in the GEE model for K-ASQ scores because the prevalence was extremely low (0.8%) in the K-ASQ responders. All statistical analysis was performed using SAS 9.4 (SAS Institute Inc.).

## Results

Mean age of mothers at the first survey was 31.3 ± 3.7 years. Majority of women were college or university graduates, unemployed at the baseline survey (Table 1). Mothers whose baby is preterm was 4.5%. The proportion of the employed among mothers increased over time. Overall, majority of women reported their monthly-average household income was below the national median level for 4-person families. Percentage of those who planned to have an additional child decreased in the fourth year. Proportion of highest quartile of parenting stress increased (*P* < 0.001). The annual prevalence of low K-ASQ score were between 0.3% and 5.3%. Prevalence of low communication, fine motor and gross motor skills increased in the third year compared to those at the baseline.

**Table 1:** Change of demographic and behavioral characteristics of 2,150 mothers of Panel Study on Korean Children, 2008–2011

Variables	2008, 1st year of survey	Weighted value
Age, mean ± SD	31.3 ± 3.7	31.4 ± 0.1
Age of partner, mean ± SD	33.9 ± 4.0	33.9 ± 0.1
Highest education		
High school or lower, N (%)	644 (30.9)	136,231 (30.9)
Highest education of partner		
High school or lower education, N (%)	636 (31.0)	136,354 (31.0)
Preterm baby, N (%)	90 (4.5)	19,705 (4.5)

Year of survey	2008	2009	2010	2011	*P* for trend^b^
	N (weighted %)	N (weighted %)	N (weighted %)	N (weighted %)	
Employed mothers	656 (31.5)	522 (27.3)	554 (30.4)	641 (35.1)	0.010
Monthly household income^a^					
< 3,442 USD	1,663 (80.8)	1,712 (77.2)	1,706 (75.2)	1,567 (69.1)	< .001
3,442–6,885 USD	377 (18.0)	354 (19.3)	385 (19.3)	525 (27.8)	0.673
> 6,885 USD	25 (1.3)	73 (3.5)	58 (3.5)	57 (3.1)	–
Plan for additional childbirth	577 (27.9)	658 (35.0)	557 (31.8)	343 (19.5)	< .001
Psychological stress related with childcare					
1st quartile	728 (34.1)	542 (28.6)	432 (24.1)	452 (25.7)	–
2nd quartile	450 (22.2)	441 (23.2)	442 (24.7)	392 (22.0)	0.001
3rd quartile	439 (21.1)	435 (23.3)	417 (22.9)	389 (22.3)	0.001
4th quartile	461 (22.6)	486 (24.9)	511 (28.4)	521 (30.0)	< .001
Low K-ASQ score^c^					
Communication	70 (3.7)	20 (1.2)	92 (5.2)	–	0.039
Fine motor skills	12 (0.4)	34 (2.0)	30 (1.7)	–	< .001
Gross motor skills	7 (0.3)	93 (5.3)	32 (2.1)	–	< 0.001
Personal and social skills	24 (1.2)	40 (2.0)	17 (1.2)	–	0.967
Problem-solving ability	34 (1.6)	45 (2.7)	28 (1.5)	–	0.931

SD, standard deviation; SE, standard error; K-ASQ, Korean-Ages & Stages Questionnaire

^a^Converted from Korean Won to U.S. dollar ($) based on the exchange rate in 2011 (1151.8 KW = 1 USD)

^b^*P* values for trend were calculated with the annual weighted prevalence

**Fig. 1:**
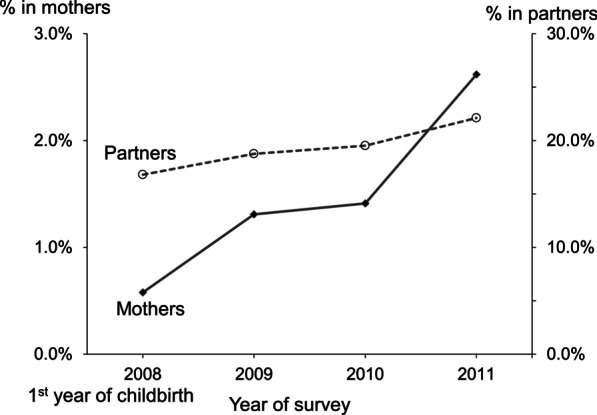
Annual weighted prevalence of harmful alcohol use among mothers (n = 2,150) and their partners (n = 1,828) of the Panel Study on Korean Children, 2008–2011

^c^Proportion of children whose score of the domain less than 2 standard deviation, higher score indicates better performance. There are 777 missing cases (12.2%). The K-ASQ was not administered in 2011. Sum of column frequency may not be equal to 1 due to missing cases. Annual prevalence of harmful alcohol use increased from 0.7% in the 1st year to 2.6% in the 4th year (*P* < 0.001 for linear trend, Fig. 1). Harmful alcohol use of their partners increased from 16.8% in the 1st year to 22.1% in the 4th year (*P* < 0.001). Prevalence of current smoking remained as low as 0.8―1.2% throughout the study period (*P* = 0.216). Similarly, the prevalence of current smoking among their partners ranged between 50.9 and 53.4% without a significant linear trend

When we analyze individual data, the odds of harmful alcohol use increased by 48% per year in mothers (adjusted OR = 1.48, 95% CI: 1.22, 1.78, Table 2). Lower education than college or university graduation was associated with higher odds of harmful alcohol use (2.52, 95% CI: 1.43, 4.45). In time-varying covariates, current smoking (7.44, 95% CI: 3.00, 18.45), harmful use of alcohol of partner (2.66, 95% CI: 1.57, 4.49) were risk factors. Odds of harmful alcohol use was higher among those employed and lower household income, which did not reach statistical significance. We did not observe an association with age at the baseline survey, preterm baby, plan for additional childbirth, or childcare stress. In partners, harmful alcohol use increased over time (1.07, 95% CI: 1.02, 1.11). This increasing trend was not evident for the annual prevalence of current smoking of partners in both mothers and their partners (data not shown). For partners, higher ORs were observed when the mothers show harmful alcohol use (1.87, 95% CI1.23, 2.84).

**Table 2:** Adjusted odds ratio of harmful alcohol use and current smoking using generalized estimating equation models, 2,150 mothers and their partners of Panel Study on Korean Children, 2008–2011

Covariates	Odds ratios (95% Confidence intervals)^b^	
	Harmful alcohol drinking	
	Mothers	Fathers
Baseline year, 2008	1.00 (reference)	
Linear effect of year	**1.48 (1.22, 1.78)**	**1.07 (1.02, 1.11)**
Age ≥ 35 years	1.18 (0.60, 2.30)	**1.28 (1.06, 1.54)**
Preterm birth	0.73 (0.16, 3.33)	0.80 (0.50, 1.29)
Highest education		
High-school or lower	**2.52 (1.43, 4.45)**	**1.26 (1.03, 1.54)**
College/University	1.00 (reference)	1.00 (reference)
*Time-varying covariates*		
Employment		
Employed	1.59 (0.97, 2.59)	–
Unemployed/housewife	1.00 (reference)	–
Monthly household income^a^		
< 3,442 USD	1.42 (0.35, 5.84)	**0.68 (0.52, 0.90)**
3,442–6,885 USD	1.22 (0.28, 5.43)	**0.65 (0.49, 0.86)**
> 6,885 USD	1.00 (reference)	1.00 (reference)
Current smoking	**7.44 (3.00, 18.45)**	**2.33 (1.97, 2.75)**
Harmful use of alcohol	–	–
Plan for childbirth	1.03 (0.60, 1.76)	–
Psychological stress related with childcare	1.25 (0.99, 1.57)	–
Harmful use of alcohol of partner	**2.66 (1.57, 4.49)**	**1.87 (1.23, 2.84)**
Smoking of partner	1.40 (0.74, 2.62)	1.10 (0.65, 1.85)

Estimates with *P* < 0.05 were bolded

^a^Converted from Korean Won to U.S. dollar ($) based on the exchange rate in 2011 (1 USD = 1151.8 KW)

^b^Calculated using generalized estimating equation models including the effect of maternal and paternal age, highest education, preterm birth, year of survey and time-varying covariates (including maternal working status, monthly household income, plan for additional childbirth, psychological stress related with childcare and partner’s harmful alcohol use)

The adjusted OR of low K-ASQ score were higher in the maternal harmful alcohol use group than in control group although the association did not reach statistical significance for all five domains (Table 3). Similarly, adjusted mean values of standardized K-ASQ score were lower when the mothers showed harmful alcohol use. There was no statistically significant difference in the mean scores of five domains between the two groups.

**Table 3:** Adjusted odds ratio (OR) of low score (< 2 SD) for each domain of the Korean-Ages & Stages Questionnaire (K-ASQ) and adjusted means of K-ASQ score, 1,891^a^ children aged 0–2 of Panel Study on Korean Children, 2008–2010

K-ASQ domain	Adjusted OR (95% CI)^b^		*P*	Adjusted mean K-ASQ score (standardized)^c^		*P* for difference
	No harmful alcohol use	Harmful alcohol use		No harmful alcohol use	Harmful alcohol use	
Communication	1.00 (reference)	1.47 (0.43, 5.08)	0.543	0.02 (− 0.04, 0.09)	− 0.18 (− 0.46, 0.10)	0.148
Fine motor skills	1.00 (reference)	2.49 (0.51, 12.07)	0.258	0.02 (− 0.05, 0.08)	− 0.22 (− 0.51, 0.06)	0.096
Gross motor skills	1.00 (reference)	1.54 (0.35, 6.73)	0.569	0.06 (0.00, 0.12)	− 0.08 (− 0.35, 0.18)	0.275
Personal and social skills	1.00 (reference)	1.48 (0.20, 11.00)	0.702	− 0.04 (− 0.11, 0.03)	− 0.27 (− 0.55, 0.02)	0.110
Problem-solving ability	1.00 (reference)	2.32 (0.51, 10.61)	0.279	− 0.02 (− 0.09, 0.05)	− 0.08 (− 0.36, 0.21)	0.680

K-ASQ, Korean-Ages & Stages Questionnaire; OR, odds ratio; CI, confidence interval

^a^There are 777 missing cases (12.2%) and the K-ASQ was not administered in 2011

^b^Calculated using generalized estimating equation models including the effect of maternal and paternal age, highest education, preterm birth, year of survey and time-varying covariates (including maternal working status, monthly household income, plan for additional childbirth, psychological stress related with childcare and partner’s harmful alcohol use)

^c^Calculated using generalized linear mixed models (GLMM). Maternal current smoking was not included in the GEE model for K-ASQ scores because the prevalence was extremely low (0.8%) in the K-ASQ population

## Discussion

We observed risk of harmful alcohol drinking consistently increases over time in mothers of under-5 children using a sample of Korean mothers observed for 4 years. Lower education, current smoking, and harmful use of alcohol of partner were risk factors for harmful alcohol use of mothers. Contrast to general expectation, association with plan for additional childbirth and psychological stress related with childcare was not evident. The association between maternal harmful alcohol use and neurodevelopmental delay of the child was generally toward positive, though it did not reach statistical significance. Using longitudinal cohort data considering the temporal change in covariates, we added knowledge of annual increase and time-dependent risk factors of harmful alcohol use in mothers of under-5 children.

The strength of our study is a population-based approach employing time-varying covariates confirming well-known risk factors for harmful alcohol use among young women [17]. Prior studies showed low income, marriage, high perceived stress, and service occupation are risk factors for harmful alcohol use in women [16, 17]. Among women with young children, we additionally observed time since childbirth and partner’s harmful alcohol use are associated with a higher risk of harmful alcohol use in mothers. In a study of biological parents of under 5 children, they observed an association between father's regular alcohol use and children's developmental delay mediated by less-skilled maternal parenting practices [13]. We postulate the increasing risk of harmful alcohol use over time can be due to their limited choice in the measures to relieve their stress, given their burden of house chores and childcare [18]. Especially for alcohol use, studies have shown gender differences in stress-related alcohol use: women are generally more likely to drink to regulate negative affect and stress reactivity while men may be more likely to drink for positive reinforcement [30].

The positive association between gender role or equality and alcohol consumption in men and women is reported in prior studies [23, 31]. In countries with higher levels of gender equality where men are more involved in childcare, men, as well as women, may be more likely to reduce their alcohol consumption when they live with children [23]. Given the high gender-based inequality in childcare in Korea [31], this also may explain the mutual increase in the prevalence in both parents in this study.

Prior studies reported maternal substance use is associated with psychological and developmental problems of their offspring [4, 13]. We observed some evidence of higher risk of delayed neurodevelopment measured by K-ASQ, though the association did not reach statistical significance. This result could be due to the low prevalence of delayed neurodevelopment in the study participants. Given the consistent pattern of higher risk of delayed neurodevelopment, we believe this finding of positive association could be replicated when the neurodevelopmental delay is more prevalent.

The finding of this study needs caution in interpretation. First, there has been no information on additional childbirths during the survey. Women who were pregnant at the time of the survey would have been less likely to use alcohol. However, based on our finding of close-to-null association between plan for additional childbirth and harmful use of alcohol, we believe the potential confounding effect in our estimation would be minimal. Second, the data on pre-pregnancy alcohol use was absent in our study which could have potentially confounded the association between harmful alcohol use of mothers and the neurodevelopment of their young children. To address the independent effect of maternal alcohol use during childcare, adjusting the association for mothers’ pre-pregnancy substance use would be important. However, our primary research question was about the patterns and risk factors of harmful alcohol use in mothers of under-5 children who are presumably motivated to change their drinking behavior. Therefore, our study finding would still have implications in providing some evidence for effective strategies for reducing harmful alcohol use in mothers.

## Conclusions

We observed the harmful alcohol use among mothers of under-5 children is associated with lower education, current maternal smoking, and harmful use of alcohol of their partner. Based on this finding, we suggest a comprehensive approach which can reduce the harmful alcohol use in both parents for more effective public health intervention during the early years of childbirth.

## Data Availability

The data used in this study is available to individual researchers or institutions upon approval of the Korea Institute of Child Care and Education (KICCE, https://kicce.re.kr).

## Abbreviations

GEE: Generalized estimating equation
PSKC: Panel Study on Korean Children
OR: Odds ratio
